# Epilepsy as a Disorder of Neuroimmune Metabolism: The Kynurenine Pathway as a Link Between Cognitive and Psychiatric Dysfunction

**DOI:** 10.3390/biom16091300

**Published:** 2026-09-08

**Authors:** Trevor W. Stone, Felix I. L. Clanchy, Richard O. Williams

**Affiliations:** Nuffield Department of Orthopaedics, Rheumatology and Musculoskeletal Disorders (NDORMS), University of Oxford, Oxford OX3 7FY, UKrichard.williams@kennedy.ox.ac.uk (R.O.W.)

**Keywords:** kynurenine, kynurenic acid, quinolinic acid, NMDA receptors, AMPA receptors, neuroimmune interface

## Abstract

In this narrative review we summarise some of the roles played by glutamate and its receptors in both epilepsy and schizophrenia, before presenting the kynurenine pathway of tryptophan metabolism as a generator of compounds acting at least partly by modulating those receptors. Increasing evidence indicates that epilepsy involves interactions between neuronal excitability, cellular metabolism and immune system signalling. The kynurenine pathway of tryptophan metabolism occupies a central position within this neuroimmune–metabolic network through its regulation of glutamatergic neurotransmission and both innate and adaptive arms of the immune response. The modulation of glutamate receptors is achieved by activating NMDA-sensitive sites by quinolinic acid and the blockade of all glutamate ionotropic receptors by kynurenic acid. In addition, emerging evidence is discussed which implicates the *GRIA3*-mediated AMPA receptor subunit GluA3 function in epilepsy and schizophrenia, and observations suggesting antagonistic actions of 3-hydroxykynurenine at kainate receptors. The evidence reviewed suggests that the modulation of the kynurenine pathway metabolism may be relevant to the development of agents which increase understanding of the mechanisms involved in epilepsy, while also addressing the psychiatric and systemic inflammatory disorders that frequently accompany it. A dysregulation of neuroimmune metabolism represents a framework for improved understanding of epilepsy and its associated neuropsychiatric comorbidities, while highlighting opportunities and challenges for therapies targeting glutamatergic and immunometabolic pathways.

## 1. Introduction

Although anti-epileptic drugs currently available are effective in many cases, disease remains largely uncontrolled in at least 30% of patients [1]. As a result, an estimated 200 potential new compounds are undergoing testing [2]. This review will summarise current knowledge of glutamate as one of the most important neurotransmitters in the Central Nervous System (CNS) likely to be involved in epilepsy and other seizure disorders and which has been a key target for new drug developments. One of the avenues being pursued is the development of antagonists at receptors for the glutamate analogue α-amino-3-hydroxyl-5-methyl-4-isoxazole-propionate (AMPA) [3,4], although there is debate on whether AMPA antagonists may be most useful for occasional drug-resistant seizures [5]. AMPAR will be given special attention here in view of the recent discovery of a specific receptor subunit in a unique class of immune system leucocytes.

As the impact of the immune system on CNS excitability, function and psycho-affective disorders becomes clearer, it is increasingly necessary to consider this immunological dimension in epilepsy [6,7], not only as a potential target which might enhance or limit the production of glutamate and the processes of glutamatergic neurotransmission, but also as a potential site of activity which is independent of CNS function but which could compromise the critical health issues of immunological innate and adaptive tolerance in so far as they affect the symptoms of epilepsy.

**Figure 1:**
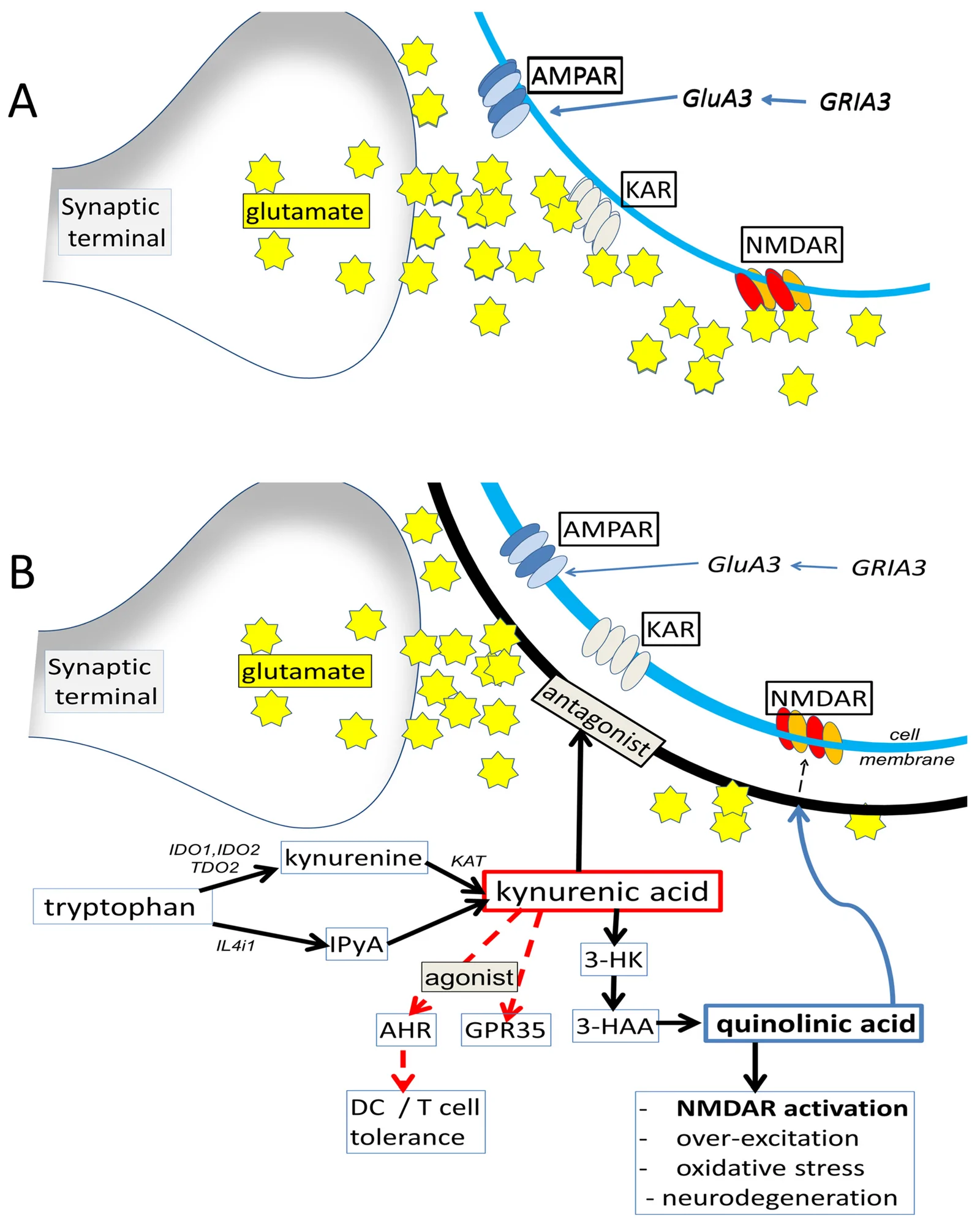
Sites and mechanisms of action of kynurenine pathway metabolites. (**A**) A synaptic terminal releases glutamate (yellow stars) into the synaptic space, from where it can activate a combination of NMDA, AMPA and kainate-sensitive receptors, each of which is composed of four subunits shown in different colours as examples One particular subunit of AMPARs indicated is GluA3 (gene *GRIA3*), which may be a factor in both epilepsy and schizophrenia, as discussed in the text. (**B**) The kynurenine pathway modulates normal activity via the production of quinolinic acid—an agonist at NMDA receptors capable of producing neural excitation and degeneration— and by kynurenic acid which blocks activation of the three receptor families. Whereas glutamate acts on all three receptor families, it is emphasised that quinolinic acid is a selective agonist for NMDARs and will be blocked by an NMDA-selective antagonist in addition to kynurenic acid. Although kynurenic acid is produced primarily by the KAT-mediated transamination of kynurenine (see Figure 2), it can also be produced by the amino acid oxidase IL4i1, via the intermediate indole-3-pyruvic acid (IPyA or I3PyA). Kynurenic acid can also activate GPR35 and the Aryl Hydrocarbon Receptor (AHR). IDO1, IDO2: indoleamine-2,3-dioxygenase 1 and 2; TDO2: tryptophan-2,3-dioxygenase; IL4i1: Interleukin-4-induced protein-1. Solid arrows indicate main metabolic routes; dashed arrows indicate alternative pathways.

The discussion will include an analysis of the role played by tryptophan metabolites in epilepsy and, in particular, in the links between epilepsy and schizo-affective disorders, especially schizophrenia. The kynurenine pathway (KP) of tryptophan metabolism generates a series of tryptophan metabolites regulating the activity of glutamate receptors [8,9,10,11], especially quinolinic acid as a selective agonist at glutamate receptors sensitive to NMDA, and kynurenic acid which is an antagonist at all three ionotropic glutamate receptors sensitive to NMDA, AMPA and kainic acid (kainate; KAR) (Figure 1). The same metabolites also determine the activity of immune system cells and their interface with neurons and glia in the CNS. This makes it important to consider the KP compounds as key mediators of neuroimmune communication having physio-pathological roles in both neurological and psychiatric maladies. Since the KP is a major factor in inflammatory activity, which contributes to the initiation and development of seizures, the pathway stands as a key molecular route linking these conditions, as discussed below.

Although the existence of a statistical relationship between epilepsy and schizophrenia is well established, the underlying reasons and clinico-pharmacological significance is not fully understood. It is in this context of accounting for two major sources of disability across the neuroimmune divide that the KP is being promoted as a critical mediator. The breadth of activity inherent in the KP may make it fundamentally relevant to even wider assessments of the relationship between epilepsy and disease: for example, the pathway has been implicated in a growing number of disparate diseases ranging from depression to cancer.

### 1.1. Glutamate in the CNS

Glutamate has been recognised as the dominant excitatory neurotransmitter in the CNS since the demonstration in vivo that an early glutamate antagonist could block an endogenous synaptic pathway in the rat brain [12]. It soon became linked to conditions in which neuronal over-excitability was a key factor, as in the epileptic disorders. Glutamate has also been considered to contribute to neurodegenerative diseases such as the dementias, Parkinson’s disease and Huntington’s disease and other neurological disorders on the basis that excessive levels of glutamate and neuronal excitability resulted in cellular calcium overload, damage and death [13]. As it became clear that there are several distinct families of glutamate receptors, the feasibility was recognised of developing pharmacological agents to manipulate them selectively in the treatment of specific CNS disorders including epilepsy.

### 1.2. Glutamate Receptors: AMPA and Kainate Receptors

The glutamate receptors linked to ion channels (‘ionotropic’ receptors) are broadly classified according to the specificity of agonists. Perhaps the most crucial glutamate receptors in the understanding of hyper-excitability and seizures are activated by AMPA and kainate respectively. These two families of receptors are responsible for the rapid synaptic depolarisation induced by glutamate. The depolarisation removes the magnesium voltage-dependent block of NMDAR for glutamate which, on a slower time scale, gives rise to the time-dependent plasticity believed to underlie learning, memory and other aspects of rapid neuronal communication including cognitive performance. AMPARs themselves are involved in receptor localisation and synaptic plasticity which are also important in these phenomena. In some cases, glutamate receptor subtypes have been linked to specific disorders such as kainate receptors in Temporal Lobe Epilepsy (TLE) [14] and AMPA receptors more generally [15]. The latter population has been particularly implicated in status epilepticus [16].

Although much effort has been placed on understanding the NMDAR in this, the initial AMPA–kainate mediated depolarisation is required and, therefore, is equally if not more suitable as a pharmacological target in cognitive disorders. This realisation has resulted in a family of AMPA-selective agonists known as ‘ampakines’ [17] which are potentially valuable in clinical treatments for cognitive dysfunction.

### 1.3. Glutamate Receptors: NMDA

The receptors sensitive to the synthetic glutamate analogue NMDA have received much attention because of their highly specific mode of activation and their unique modulation of excitability. They are unusual receptors in requiring two different agonists for activation. Glutamate itself binds primarily to the GluN2 subunit of the tetrameric NMDA receptors (NMDAR), while the co-agonist is glycine which binds to the Gly-2 (or strychnine-resistant glycine receptor) on the GluN1 subunit which is essential for full receptor activation [18]. Blocking either site by a selective antagonist is sufficient to prevent receptor activation. It is important to note that the need for two independent ligands for full NMDAR activation is a factor in explaining why kynurenic acid is a more potent antagonist of NMDAR than AMPAR or KARs. Although the difference in the potency of kynurenic acid is not great (IC50 for kynurenic acid at the glycine-B site is approximately 43 μM, whereas it is 140 μM at the glutamate binding site) [19], that alone should not affect overall potency. However, since co-activation is needed, any interference at either site by kynurenic acid will have a disproportionate influence on receptor activity.

The NMDA receptors have been of major interest because their associated ion channels are normally largely blocked by magnesium ions. The action of synaptically released glutamate is to bind to the AMPA and kainate receptors and produce a rapid depolarisation as noted above, and that in turn reduces the magnesium blockade of the NMDA receptor ion channels. The actual depolarisation by NMDA receptors, therefore, is delayed beyond the start of action of the AMPA and kainate receptors. In contrast to the few milliseconds’ duration of the AMPA/kainate response, the effects of NMDA can occupy tens or hundreds of milliseconds or more. The importance of this is that the longer response time allows sufficient calcium to enter a neuron to enable modulation of biochemical pathways capable of sustaining long-lasting changes in excitability. Those changes—such as LTP and LTD—probably underlie many aspects of cognitive behaviour. The NMDA receptors also play significant roles in the development of epileptic seizures [20,21], and may determine the threshold for repeated seizures as seen in animal models.

### 1.4. Glutamate and the Immune System

Although epileptic disorders are considered primarily a dysfunction of neural circuitry and excitability in the CNS, there is much to suggest that the immune system plays a significant role in the initiation and progression of the illness and related disorders [22,23]. Part of this involvement stems from the movement of immune system leucocytes into the CNS parenchyma, and partly from the increased penetration or endogenous generation of inflammatory mediators such as the interleukins and chemokines [24], or members of the Tumour Necrosis Factor-α (TNF-α) superfamily [25,26] across the blood–brain barrier, with the potent pro-inflammatory cytokine IL-6 playing a major role [27]. These mediators can affect the release of glutamate and its activation of ionotropic receptors [28,29]. Initial epileptic attacks can increase blood–brain barrier permeability for extended periods, potentially contributing to the drug resistance which can develop rapidly [30,31,32].

In parallel with these developments there has been growing interest in the extra-neural properties of glutamate, with several reports on the existence of functional glutamate receptors on various populations of leucocytes in the immune system. It is rare to find two studies with comparable results on the potency, selectivity and functional sensitivity to glutamate and its related ligands, but a recent review of the literature and a collation of >25 gene expression studies concluded that the level of gene expression of glutamate receptors (AMPAR, NMDAR, KAR and metabotropic receptors) on primary cells (in health and disease) was at the limit of detection and certainly lower than published levels of protein expression would indicate [33]. Nevertheless, the presence of even minute amounts of receptor could mean that they contribute to immune cell function, albeit under very limited conditions of stimulation or activation. In particular, they could be involved in wider activities of the immune system, especially its role in communication between cells of the immune system and CNS (the ‘neuroimmune interface’), and in the initiation and progression of chronic disorders such as those neurological and psychiatric conditions being discussed here.

## 2. The Neuroimmune Interface

This term refers to the crosstalk between cells of the immune system and cells of the CNS, mediated partly by a plethora of secreted molecules and their receptors. These include the cytokine and chemokine families [34,35]. TNF-α is also relevant, since it markedly influences synaptic efficacy by up-regulating surface expression of AMPA receptors via its TNFR1 receptor influencing the phosphatidylinositol-3 (PI3) kinase-dependent processes. TNF-α also decreases synaptic inhibition via the endocytosis of GABA-A receptors [36].

These and many other aspects of the neuroimmune interface are highly relevant to the understanding and treatment of epileptic disorders. TLE is a chronic, often drug-resistant form of epilepsy [37,38] in which cognitive decline is an inevitable component. It is a good example of the results of an increased level of pro-inflammatory mediators such as interleukin-1β [39]. Indeed, epileptic attacks are strongly associated with cognitive dysfunction [22,40,41], potentially involving Toll-Like Receptors such as TLR2 and TLR4 which play significant roles in cognition function [42] as well as contributing to Post-Operative Cognitive Dysfunction through the induction of neuroinflammation [43]. HMGB1 (High Mobility Group Box-1) impairs memory by mediating the effects of Receptors for Glycation End-products (RAGE) and TLR4 [44]. Cognitive dysfunction can be ameliorated by inhibiting hippocampal TLR4 activation in aged rats [45].

In addition to the movement of leucocytes into the CNS, a local inflammatory milieu can be achieved by the glial cells. Precursors to microglia enter the CNS early in development. As a bi-directional system, the neuroimmune interface is responsible for the communication not only in the sense that immune system activation or modulation can affect neuroglial function in the CNS, but also the converse sense [46]. Microglia in particular are tightly linked to neuronal activity, with their metabolism sensitive to neuronally mediated changes in the chemical composition of the extracellular medium. Among the many factors released from microglia and, to some extent the astrocytes, are interleukins and chemokines able to regulate specific features of blood–brain barrier permeability, and powerful inflammatory agents such as TNF-α [47].

### Glutamate Receptors as Drug Targets

The link between glutamate neurotransmission and epilepsy is now well established, most notably by the ability of glutamate antagonists far more potent and selective than the original compounds, to reduce neuronal hyper-excitability and to control the number and severity of seizures [14]. One of the first in a new class of compounds was the non-competitive AMPAR antagonist perampanel [48,49,50,51,52] followed by related drugs such as telampanel and selurampanel [53,54,55,56].

Like many anticonvulsant drugs, the perampanel family are effective in inhibiting action potential burst firing [52]. These have added to a long list of drugs having value in treating epilepsy although most are effective in different subgroups of patients (for example: topiramate, felbamate, pregabalin, riluzole, memantine).

The extent of involvement of the glutamatergic systems in epilepsy has been emphasised by the finding that the postsynaptic receptors are not the only potential molecular targets. Simply interfering with the uptake and removal of extracellular glutamate, which is normally cleared by membrane and vesicular transporters, is sufficient to alter neuronal excitability and to control the parameters of seizure disorders [57,58,59,60]. As a consequence, one therapeutic implication of these observations is that glutamate transporters may provide alternative targets for controlling neuronal excitability when direct modulation of glutamate receptors is difficult or ineffective since the structure–activity restrictions on transporters are generally less stringent than needed for receptor modulation. With increasing genetic information on receptor polymorphisms and mutations it should be possible to target those selective elements of molecular structure and function pharmacologically, or to modify the abnormal genes directly. In that context, recent observations on AMPAR subunits may be highly relevant.

## 3. AMPAR Subunits: A New Dimension

A recent discovery has opened a new window on understanding the neuroimmune interface to a level at which it may explain a common origin of several superficially distinct disorders including epilepsy. Both AMPA and kainate receptors are composed of four subunits, generating receptor populations of significantly different sensitivity to glutamate and different ion channel kinetics. The rates of depolarisation and re-polarisation, and of de-sensitisation and recovery, for example, are also distinctly different. Consequently, any change in the absolute or relative quantities of the AMPAR subunit GluA1-4 will significantly affect the properties of the affected neurons. The absence, overexpression or mutation of a subunit could have a marked influence on excitability and plasticity, contributing to chronic disorders such as epilepsy and schizophrenia and potentially explaining the well-documented association between epilepsy and cognitive performance.

During the examination of glutamate receptor presence and quantification in a wide range of leucocytes, it was discovered that one of the AMPAR subunit genes—*GRIA3*—was localised alone rather than complexed with other subunits—in a small population of leucocytes [61], *GRIA3* codes for one of the normally hetero-tetrameric AMPAR subunit proteins: GluA3. The cells expressing *GRIA3* in isolation are plasmacytoid dendritic cells (pDCs) which make up only approximately 0.5% of the total leucocyte pool, but they are among the first cells in the immune system to be activated by viral infection. Viral nucleic acids activate TLRs in the pDCs, especially TLR7 and TLR9, which then promote the rapid synthesis and secretion of Type I interferons (α- and β-). Together with the proliferation of classical cDCs generated by pDCs, this rapidly spreads to cause the organism-wide, generalised, response to the virus.

The discovery of this gene for the AMPA subunit GluA3 raises the possibility that gene or protein transfer might occur between the activated pDCs and neurons or glia in the CNS. This mechanism, which could involve intercellular molecular movements in a variety of extracellular vesicles, has been presented and discussed in detail elsewhere [61]. This situation would be particularly relevant in view of the KP activation induced by infections, inflammation and various forms of stress, since kynurenic acid is a known antagonist at AMPARs. Although not yet tested experimentally, it is likely that the different AMPAR subunit combinations—with or without a GluA3 component—would have a different sensitivity to kynurenic acid, so this would compound the differences in cell properties resulting from the shift in AMPAR composition. Since part of the biological activity of kynurenic acid is thought to be the provision of an antagonistic balance to the deleterious effects of quinolinic acid on NMDARs, one result would be an altered tendency for cell depolarisation and neurotoxicity. The same change in excitatory drive—increased or decreased—could also compromise the normal organisation of neural network activity, leading to interference with cognitive processes.

In young mammals (including humans) in which the CNS is still in early stages of development, there will be a substantial risk that the proliferation and growth of neurons and glia—many aspects of which are dependent on glutamate receptors—will be affected, potentially leading to the emergence of disease in post-natal and adolescent or adult life. Studies in rodents have demonstrated that altering KP activity during gestation or early post-natal life, can affect the structure, neurochemistry and electrophysiology of the CNS in adulthood, with behavioural changes being reported, as discussed below.

### Glutamate and the Epilepsy-Schizophrenia Link

Changes in the level of neuronal excitability will not only be directly relevant to conditions such as epilepsy, but will have far wider implications, since any change in excitability of one neuronal pathway will disrupt the temporal precision of wider integrated neural networks (Figure 1). This may explain why glutamate is important not only in physical, neuromuscular activity and disorders, but is also crucial in the finely tuned neuronal interactions that underlie learning, rational thought and executive function.

Schizophrenia is one example of CNS disorders in which the primary dysfunction is in the processing and integration of sensory and internal neural networks. This leads to difficulties in learning and memory but, in particular, problems with logical and rational thought. Altered perception and cognition can contribute to delusions, hallucinations and abnormal behaviour. A key development, therefore, was the realisation that glutamate antagonists such as ketamine or dizocilpine (MK-801) administered chronically to rodents, could induce signs of behavioural traits resembling symptoms of schizophrenia. They included alterations of pre-pulse inhibition, a cardinal sign of psychosis in human patients in addition to experimental animals [62,63]. Overall, the current view of the cause of these psycho-affective conditions has been encapsulated in the ‘hypoglutamatergic hypothesis’ that symptoms are the result of reduced glutamatergic activity. For that reason, interest in details of the nature of that glutamate dysfunction has been increasing, with the development of the ampakine agonists [17].

Initially, there appear to be few mechanistic links between epilepsy and psychiatric disorders such as schizophrenia, major depression and autism spectrum disorders. However, increasing evidence indicates that activity of the immune system contributes to the symptoms and progression of schizophrenia [64] and, conversely, epilepsy has been linked to significant cognitive dysfunction [65].

The relationship between epilepsy and schizophrenia has long been an area of controversy, since the epidemiological links between them were not readily comparable with knowledge of the underlying biology [66]. As a result, various proposals for neurotransmitter involvement in schizo-affective disorders have been made, especially where they interact with immune and inflammatory conditions [22,64]. Nevertheless, the overlap between epileptic states, autism and schizophrenia is emphasised by the ability of some glutamate antagonists to ameliorate both seizure severity and symptoms of these disorders [67,68]. With these acknowledged relationships between epileptic syndromes and psychiatric conditions such as schizophrenia and autism, it is intriguing that the same molecules—the GluA3 AMPAR subunit of AMPARs and the tryptophan metabolite kynurenic acid discussed below—have been linked to both these disorders [69,70] raising the possibility of a rational molecular explanation for their relationship: these apparently distinct neurological and psychiatric conditions may share common molecular mechanisms related to glutamatergic signalling and neuroimmune regulation. It is consistent with these arguments and studies of genetic polymorphisms that these can contribute to the disorders under consideration [70,71,72,73,74], including many with clear associations with epilepsy [75,76,77,78]. The relevance is also supported by strong links between neuropsychiatric disorders and the presence of GluA3 antibodies [71].

A key role for AMPAR and GluA3 in particular is supported by the proposed role of kynurenic acid in schizophrenia. In view of the poor efficacy and the chronic occurrence of adverse effects associated with classical antipsychotic drugs, alternative concepts were sought by testing for potential biomarkers which might reflect a loss of glutamate-dependent synaptic transmission. This approach led to the finding of increased levels of kynurenic acid in the cerebrospinal fluid (CSF) of patients with schizophrenia, observations which emphasise a significant link between disorders of the immune system and CNS [79,80,81,82]. Levels of kynurenic acid in the CSF or brain tissue are increased in patients with schizophrenia [80,81,82,83]. Supporting evidence for these assertions have been derived from meta-analyses [84] and genetic studies including the presence and effects of polymorphisms [85,86,87]. The raised levels of kynurenic acid contribute to the loss of synaptic function [88] and are not the result of antipsychotic drugs use [89]. In addition, reducing CNS kynurenic acid concentrations enhances cognitive function [90]. When considering the significance of these tissue concentrations, however, it is essential to recognise that comparability between the different fluids is heavily dependent on the respective rates of generation and metabolism, together with the limiting permeability of the blood–brain barrier to many polar compounds.

As will be clear from the above discussion, the KP of tryptophan metabolism holds a potentially key place in the understanding and possible therapeutic developments in both schizo-affective disorders and epilepsy, so it is appropriate to consider the pathway in more detail in the following sections.

## 4. The Kynurenine Pathway (KP)

**Figure 2:**
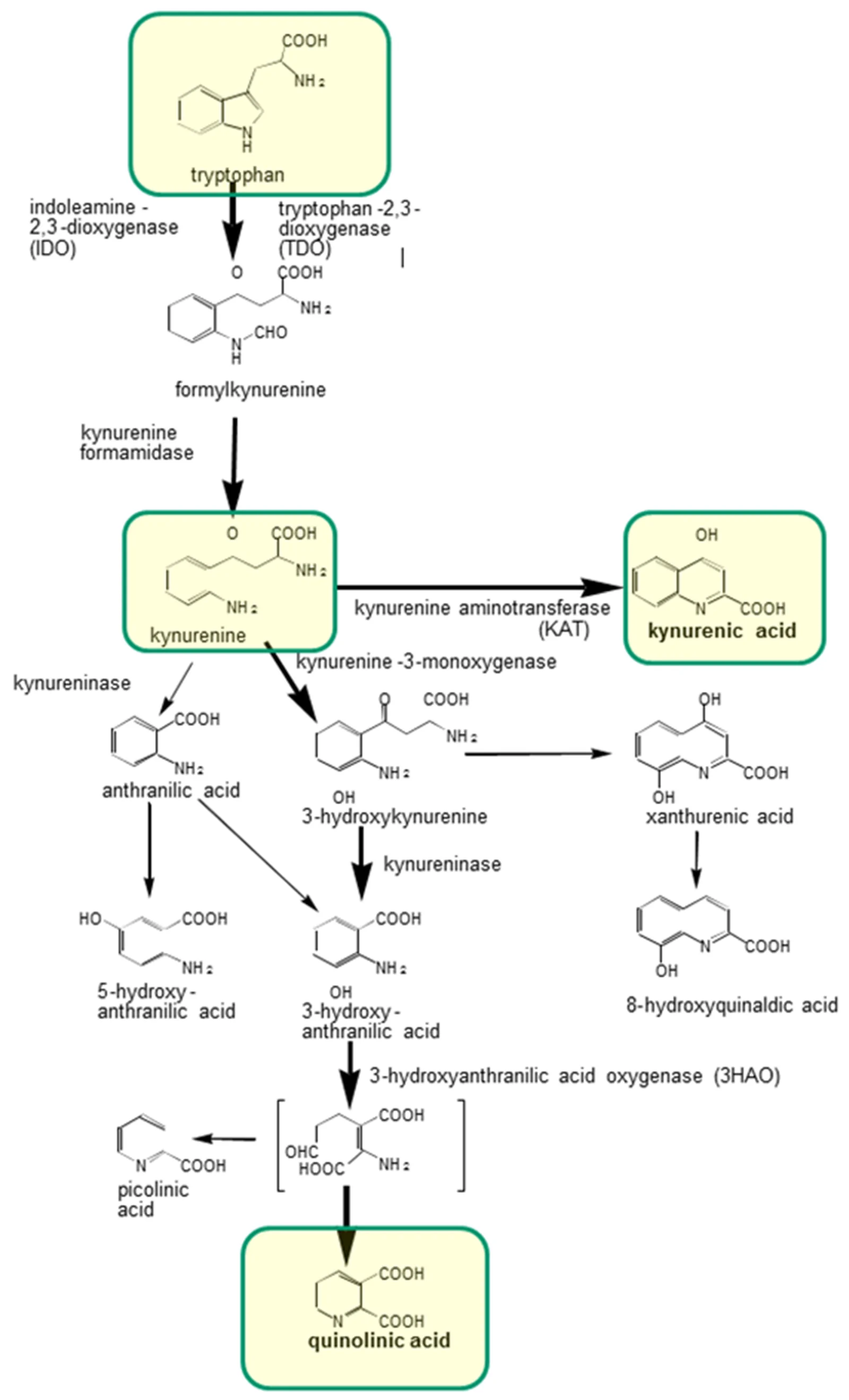
The major elements of the kynurenine pathway. The pathway involves the oxidation of tryptophan to kynurenine, which is further oxidised via kynurenine-3- mono-oxygenase to 3-hydroxy-kynurenine and thence to quinolinic acid, an agonist at NMDA receptors. Kynurenine is also transaminated to kynurenic acid which blocks ionotropic glutamate receptors sensitive to NMDA, AMPA or kainate.

**Figure 3:**
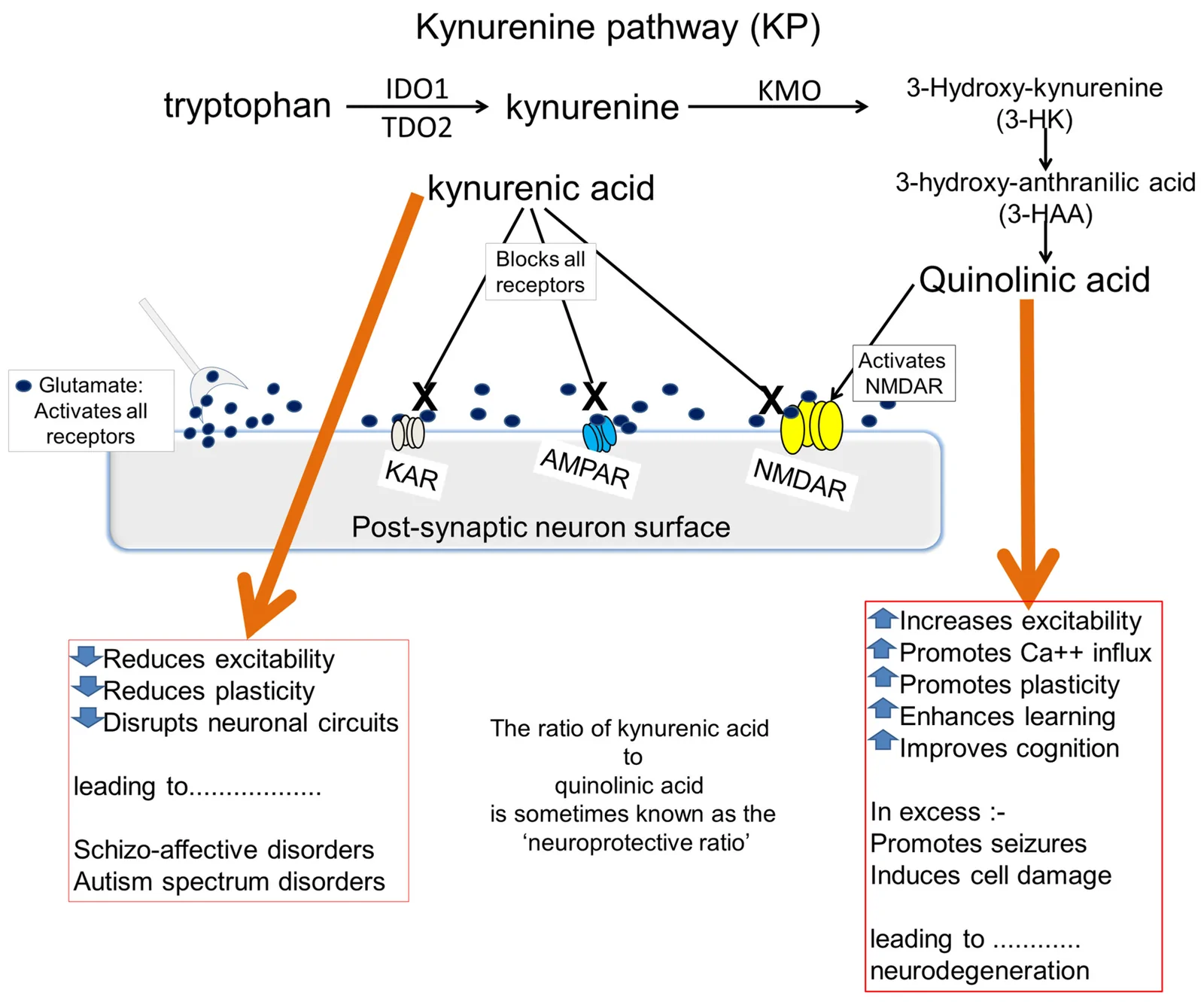
The glutamate receptor actions and clinical implications of the kynurenine pathway. The main elements of the kynurenine pathway are shown, acting on the ionotropic glutamate receptors, indicating their primary actions and their links to the clinical disorders of epilepsy and schizophrenia. Their overall effects are likely to depend on the relative concentrations of glutamate, quinolinic acid and kynurenic acid. Kynurenine can be transported from the intracellular pathway to other cells. Alternative receptors for kynurenic acid such as AHR and GPR35 will also influence their biological activities. Primary location of the pathway is intracellular (yellow), with major receptors on post-synaptic structures (blue) Red arrow and boxes summarise biological activity.

The KP has been introduced above in view of its key roles in the modulation of glutamatergic synaptic transmission. However, there are wider implications which are relevant to an understanding of neurodevelopmental disorders, acute and chronic. Although often overlooked compared with the serotonin (5-hydroxytryptamine, 5HT) pathway, tryptophan oxidation is primarily mediated by its conversion to kynurenine and subsequent compounds which account for the metabolism of around 95% of free (non-protein) tryptophan. A schematic summary of the pathway (Figure 2) indicates the generation of several substances with significant biological activity, while Figure 3 relates these to clinical disorders. As introduced above, the first metabolite found to have pharmacological activity was quinolinic acid which was a selective agonist at the NMDAR in rat brain in vivo [91]. Pursuing the implied molecular structural similarity between quinolinic acid and NMDA, other compounds in the same metabolic pathway from tryptophan were then examined, leading to the discovery of kynurenic acid. This is a transamination product of kynurenine, which proved to be an antagonist at all three types of glutamate receptor, sensitive to NMDA, kainate or quisqualate (an early agonist for AMPA receptor) respectively [8,9,92]. This compound rapidly became a standard laboratory tool for the identification of glutamate-releasing neuronal pathways, with a substantial development of derived analogues and derivatives for potential clinical use [93]. Later work showed that kynurenic acid blocked not only the glutamate binding site of NMDARs, but also the co-agonist glycine binding site [94] and it was soon recognised as a key endogenous metabolite able to block both glutamatergic synaptic transmission and excitotoxic neuronal damage [8]. The primary relationship between KP metabolism and the generation of seizures is based on the concept that it is the ratio between NMDAR activation by quinolinic acid [91] and kynurenic acid as a glutamate antagonist (Figure 3) [92]. This dual interference led to the concept that modulating that ratio by inhibiting kynurenine-3-mono-oxygenase (KMO) (Figure 2) thus increasing kynurenic acid levels and reducing quinolinic acid may be a valuable approach to the treatment of epilepsy and some neurodegenerative disorders [95,96].

Somewhat later, it was observed that interfering with the kynurenine pathway could alter cell and embryonic survival, leading to studies exploring its possible role in the immune system. One of the most influential studies of this kind was the demonstration that increasing kynurenic acid concentrations in gestating rats by inhibiting KMO resulted in a marked loss of embryo viability [97]. This observation effectively introduced the development of work on the kynurenine pathway in the immune system. The parallel activity of the pathway on neuronal function and immune cells identified it as a major player in neuroimmune communication, a concept which is continually being expanded by new information and conceptual advances [10,98]. In view of its activation by inflammatory mediators, especially IFN-γ, it has recently been suggested that the kynurenine pathway could function as an organism-wide compensatory response to infection, inflammation and other forms of stress, in the manner of a reflex feedback system [10,11]. This view would place a great deal of importance on the pathway and its physiological and pathological activities and, of course, its potential for therapeutic modulation. The complex interplay between the kynurenine pathway and immune system mediators has been described in some detail [10,11].

As the wider actions of kynurenic acid on immune tolerance, cognitive function, tissue regeneration and other effects were examined in a variety of experimental conditions, several possible alternative targets for kynurenic acid were identified. One is the G-Protein coupled Recepor-35 (GPR35) [99] where kynurenic acid remains one of few endogenous compounds able to activate the site. An additional target was discovered while studying the xenobiotic sensor Aryl Hydrocarbon Receptor (AHR) [100]. The AHR was reportedly activated by kynurenine, although later work has indicated that kynurenic acid is also an agonist. In many situations it remains unclear whether kynurenine or kynurenic acid is the primary activator, or whether (as seems likely), kynurenine is primarily a precursor for active kynurenic acid. It is of interest that the most potent agonist known (6-Formylindolo [3,2-*b*]carbazole, FICZ) is a photo-oxidation product from tryptophan, emphasising the key position which this essential amino acid holds in biology. The AHR remains a prime objective of immunological research as it is a pivotal factor in the modulation of innate and adaptive immunity. It is a central element of the kynurenine pathway not only from its sensitivity to kynurenine or kynurenic acid as ligands, but also because it regulates the production of IL-6, an important, positive, feedback activator of IDO1 [101,102].

### 4.1. Indoleamine-2,3-Dioxygenase-1 (IDO1)

As an introduction to this section, it is important to note the variety of methods used to quantify IDO and its activity. It is a common fallacy that measurement of the ratio between tryptophan and kynurenine concentrations provides an adequate assessment of IDO enzymic activity; this is rarely correct since both compounds are obtained from a variety of source. Tryptophan is present in the diet and is both synthesised and metabolised by the microbiome, and is metabolised by mammalian enzymes such as the tryptophan hydroxylases and interleukin-4-induced protein-1 (IL4i1). Amino acid transporters including those for tryptophan and kynurenines (such as the Large Amino Transporter1, LAT-1) also contribute to the regulation of tryptophan and kynurenine levels. As we have emphasised recently [10,11] the only useful method for enzyme activity quantification is using ELISAs to measure substrate and product concentrations under clearly defined conditions.

As the main enzyme initiating the KP, IDO1 has been a target for drug developments in cancer and autoimmune disorders, but with its metabolic products having the range of physiological activity summarised above, it is increasingly attracting attention as a potential mediator in epilepsy. The evidence linking IDO directly with seizures is controversial. While some virally induced episodes are increased by IDO1 deletion [103], other models exhibit a suppression of seizures [104,105]. Since a loss of IDO1 will alter the balance between pro-convulsant quinolinic acid and anticonvulsant kynurenic acid, more work on the downstream enzymes such as KMO, KATs and kynureninase will be needed to clarify the reasons for these differences.

Temporal Lobe Epilepsy (TLE) is associated with a number of metabolic factors including the Brain-Derived Neurotrophic Factor (BDNF) and TrkB pathways, oxidative stress and mitochondrial dysfunction, all of which can be enhanced by activation of IDO1 [106]. This is partly due to the high redox activity of the downstream kynurenine metabolites 3-hydroxy-kynurenine (3-HK) and 3-hydroxyanthranilic acid (3-HAA) [107,108]. This raises the possibility that IDO1 inhibitors might be beneficial in TLE. Indeed, the levels of kynurenine and its metabolites are frequently abnormal in the brain or CSF of patients with TLE as well as in experimental models [109], raising the possibility of a causal association with seizure activity [110] and the underlying neuronal overactivity [111]. Predictably, the concentrations of kynurenine and kynurenic acid were reduced in patients with drug-resistant epilepsy, correlating with seizure frequency during multivitamin supplementation [112].

In a combined study of patients with epilepsy and mice with seizures induced by lithium and pilocarpine, the kynurenine: tryptophan ratio (a measure of IDO activity) was increased in the CSF, along with high levels of inflammatory cytokines [104,113]. Importantly, seizures were attenuated in IDO1-deficient epileptic mice, which was accompanied by a reduced level of the NMDAR agonist quinolinic acid. This probably contributed to the reduced seizure activity and also accounts for the enhanced neuronal viability observed. Both kynurenic acid and a synthetic analogue, SZR104, have anti-inflammatory activity reflected in their inhibition of microglial activation after status epilepticus [114]. As a result, the combination of IDO1 inhibitors with conventional anticonvulsants such as valproate, which itself remain ineffective in many patients, have been advanced as valuable treatments [115]. In the search for improvements in IDO1 regulation, it has been reported that there are important differences between IDO1 and IDO2 with respect to their modulation of seizure activity in the kainic acid-induced model of epilepsy [116]. Nevertheless, a cell-type selective modulation of these enzymes was considered a more targeted approach to controlling seizures.

Other conditions have been noted on similar lines, such as the apparent paradox that an increase in virally induced seizures in mice resulting from IDO1 deletion [117]. Notably, this was not accompanied by any change in inflammatory cytokine levels but was associated with enhanced hippocampal damage. This may have been a result, at least in part, of the reduced kynurenic acid production which could have increased the quinolinic acid: kynurenic acid ratio to the more toxic level at which the activation of NMDARs was raised relative to control values.

### 4.2. Kynurenine-3-Mono-Oxygenase (KMO)

Alternatively, downstream enzymes may be even more useful. Inhibiting KMO has, in principle, the effect of reducing quinolinic acid synthesis and simultaneously raising the conversion of kynurenine to kynurenic acid [118]. However, the potentially deleterious effect of kynurenic acid on cognition, for which inhibitors of kynurenine transaminases are actively being developed, requires further experimental investigation.

Given that kynurenic acid or the kynurenic acid: quinolinic acid ratio are probably the primary factors responsible for anticonvulsant activity, it may not be a coincidence that activation of GPR35—noted above as a membrane protein activated by kynurenic acid [99]—can depress epileptic seizures and neuronal overactivity [119]. It may be that several active drugs such as levetiracetam may work by a combination of factors which include glutamate antagonism at AMPARs and modulation of KP to inhibit quinolinic acid generation together with an enhancement of kynurenic acid levels [120]. It is also possible that the reputedly beneficial effects of the ketogenic diet (high fat, low carbohydrate) [121] are mediated by the resulting changes in kynurenine pathway metabolites [122] especially in cases of refractory epilepsy [123,124]. The fundamental question of how the changes are induced by the diet remain unclear [121,125].

It is important to note the differential localisation of the KP downstream enzymes within the CNS. Expression of the full pathway (Figure 2) in microglia allows the generation of quinolinic acid [126]. Since microglia are developmentally derived from—and related to—immune system macrophage cells, they are substantially involved in the tissue response to local damage and infection Their activation by inflammatory cytokines promotes activation of the KP and the generation of quinolinic acid, potentially explaining the neuronally damaging effects of systemic inflammation. Astrocytes, however, express only KAT, not KMO, allowing the generation of kynurenic acid but not quinolinic acid [127]. The relative state of these major glial families, therefore, is likely to be a pivotal factor in the production of CNS inflammation and related disorders.

### 4.3. 3-Hydroxykynurenine (3-HK)

Kainic acid is the most potent excitatory compound at glutamate receptors, presenting KARs as a potentially important drug target. Certainly, KAR activation is able to generate seizures, and the kainate model of seizure activity is popular experimentally. Kainate also produces neuronal damage and neuropathological lesions similar to those found in TLE for which is a useful model. Although not usually considered as a receptor-mediated ligand, the kynurenine metabolite, 3-hydroxykynurenine (3-HK; Figure 2) has been shown to act on immune system cells in a zebrafish model, apparently blocking a kainate-like receptor to promote resistance to bacterial infection [128]. If 3-HK is also able to block neuronal KARs, it may be possible to develop analogues with the ability to reduce excitability and the burst firing which underlies seizures and epilepsy.

### 4.4. Neurodevelopmental Disorders

One aspect of the two areas of epilepsy and schizophrenia which require special attention is the progression of symptoms and the development of drug resistance in some patients [129,130]. Indeed, in both of these conditions, only a minority of patients experience substantial and maintained relief from using the standard prescription drugs. While this situation has not been fully explained, it is possible that it reflects the progressive change in a metabolic system which modulates glutamate receptor function, such as the KP.

When the KP is activated as part of the immunological response, the early driving force is to the formation of kynurenine and its immediate metabolites kynurenic acid (via kynurenine aminotransferase, KAT) or 3HK (via KMO) (Figure 2). Kynureninase is also active in the conversion of kynurenine to anthranilic acid, although this arm of the KP is less understood because two isozymes are present—kynureninase-1 producing 3-HAA and kynureninase-2 producing anthranilate—and the factors affecting these two enzymes and their relative activities are not entirely clear. This is partly because anthranilic acid is a prominent factor in microbial metabolism as the initial compound being converted to tryptophan. This pathway and their impact on the KP in the host tissues is increasingly recognised as an important influence on host biology which could influence the development, progression and drug sensitivity of the disorders under consideration [131,132]. However, although the microbiome generates several bio-active indole-derived compounds, we have not included this topic here. The primary concern is that of biochemistry and metabolism with directly relevant actions in the CNS, irrespective of the origin of the KP components.

However, as activation of the KP develops, the more proximal enzymes will become progressively saturated and more kynurenines will be metabolised to 3-HAA and quinolinic acid. Since quinolinic acid is excitatory, acting selectively as an agonist at NMDARs, it is likely to reach a concentration that can cause over-excitation, cell damage and degeneration.

As noted above, early CNS development is heavily dependent on glutamatergic neurotransmission. These will be influenced by activity in the KP potentially resulting in the emergence of disease in post-natal and adolescent or adult life. Interference with the pathway during gestation or neonatal life has been shown to affect CNS structure, biochemistry and electrophysiology as the former embryos and neonates reach adulthood [133,134] with behavioural changes being reported [135,136]. Conversely, reducing CNS kynurenic acid levels by inhibiting KAT could prevent abnormal behaviours induced by other neurodevelopmental procedures [137,138]. Exposure of the pregnant female to excessive or prolonged stress—physical or emotional—could induce the KP and such aberrations.

### 4.5. Chronic Diseases

In addition to providing an explanation of neurodevelopmental disorders, another possible relevance of these issues is in the progression and treatment of disease. In some patients, neurological disorders such as epilepsy can progress from an early, relatively mild and reversible stage, to a drug-resistant phase which may depend on NMDARs as seen in animal models of seizure kindling. Indeed, in many of these conditions, only a minority of patients experience substantial and maintained relief from using the standard prescription drugs. While this situation has not been fully explained, it is possible that the failure of current therapies reflects their limited or insufficient targeting. It is possible that the KP is one of the potential targets which should be considered (Figure 3).

### 4.6. Kynurenine Metabolites as Biomarkers

One of the major problems in clinical diagnosis and management is obtaining an accurate assessment of symptoms which often vary between individuals with similar, or related, medical conditions. To improve the value and reliability of those assessments, some form of objective, quantifiable measure of the differences between diseases and patients is increasingly recognised as essential. To that end, much work is devoted to identifying which compounds are most appropriate for different disorders. The KP is proving highly relevant in this respect, as it changes with the degree of neural or immune system activity and can therefore reflect essential disease parameters such as cognitive dysfunction and immune system function. As a result, the level of KP compounds has been used in the assessment of cognitive decline in a range of inflammatory disorders, where they reflect a composite of glial activation, blood–brain barrier permeability, cytokine activity and other factors [139]. Kynurenine metabolites have been linked to cognitive deficiency in cases as varied as post-surgical trauma [140], renal disease [141] and childhood malnutrition [142].

In the areas of interest here, KP compounds are of substantial interest in the understanding of epilepsy [143], and levels in the CSF have been found valuable as biomarkers of the presence and severity of infantile epileptic seizures [144]. They have been recommended also as markers of disorders involving slow deterioration of the CNS. For example, they allow an objective quantification of the state and rate of progression of multiple sclerosis [145] and the intensity of chronic pain [146]. Indeed cognitive dysfunction is often associated with motor difficulties in Alzheimer’s disease [143], where KP activity appears to correlate highly with many of the other molecular markers described in that disorder [147,148]. The diagnosis of schizophrenia, its progression and response to drug treatments is often contentious, but as a disorder which encompasses cognitive dysfunction without overt dementia, it is proving especially amenable to assistance from biomarkers in the KP [149,150]. KP estimation is often considered with other inflammatory pathways such as those involving NFkB, MAP kinases and inflammasomes [151] although the KP has been a focus for the assessment of disorders in which the AHR—one of the main targets of kynurenine and kynurenic acid—is considered pivotal [152].

Thus, the KP is of growing interest for biomarkers, although there are significant doubts on whether measurements in peripheral tissues—including blood—are as meaningful or helpful as those using CSF. To some extent, this will clearly depend on the central or peripheral nature of the disorders under discussion, but there is a growing recognition that, although direct measurements in the CSF are preferable, even peripheral blood levels can provide valuable information [152].

## 5. Summary and Conclusions

Glutamic acid is the dominant excitatory neurotransmitter in the mammalian CNS. It is therefore fundamental to almost all aspects of life, and consequently a potential cause of disorders of many kinds, including many neurological and psychiatric conditions. These usually include disorders of neuronal hyper-excitability such as the epileptic disorders and neurodegenerative diseases. However, since glutamatergic neurotransmission can be modulated by products of tryptophan metabolism along the kynurenine pathway, this route provides a means of understanding acute and chronic aspects of these disorders, and should be considered as a valuable potential target for drug development.

With several enzymes in the pathway, a greater analysis of structural and genetic variations, and of the potency and specificity of modulators (inhibiting or enhancing activity) should be strongly encouraged. Several aspects of cell metabolism are discussed in relation to their role in epilepsy, including the role of AMPAR subunits especially the *GRIA3* gene and GluA3 protein. While the idea of GluA3 as a possible common factor in epilepsy and schizophrenia is attractive, it remains a matter of speculation which requires experimental validation. New insights are introduced into the pharmacological potential of the KP and the need to retain pharmacological balance between the needs to reduce neuronal excitability for anti-epileptic activity and the need to enhance glutamate receptor activity to oppose schizo-affective disorders. This accounts for the expanding interest in the KP which provides a crucial functional bridge between the immune system and CNS. It also introduces a variety of molecular targets with which to approach disorders of neuronal excitability including epilepsy and associated cognitive dysfunctions such as the schizo-affective conditions. The KP is a central neuroimmune–metabolic regulator that may contribute to both seizures and their psychiatric comorbidities, possibly providing new therapeutic opportunities.

## Data Availability

No new data were created or analysed in this study. Data sharing is not applicable to this article.
